# Hospital characteristics preferred by medical students for their residency programs: A nationwide matching data analysis

**DOI:** 10.1002/jgf2.370

**Published:** 2020-08-26

**Authors:** Yuji Nishizaki, Rieko Ueda, Tomohiro Shinozaki, Yasuharu Tokuda

**Affiliations:** ^1^ Medical Technology Innovation Center Juntendo University Tokyo Japan; ^2^ Department of Cardiovascular Biology and Medicine Juntendo University Graduate School of Medicine Tokyo Japan; ^3^ Department of Information and Computer Technology Faculty of Engineering Tokyo University of Science Tokyo Japan; ^4^ Department of Medicine Muribushi Okinawa for Teaching Hospitals Okinawa Japan

**Keywords:** emergency care, matching program, medical student, primary care, resident physician, super‐rotation curriculum

## Abstract

**Background:**

In 2004, Japan introduced a mandatory 2‐year postgraduate training program for graduating medical students with a super‐rotation curriculum. A national matching system was established to determine the hospital residency programs best suited for the students. We examined the hospital characteristics preferred by applicants for residencies.

**Methods:**

A nationwide cross‐sectional study was conducted. Data on salaries, bonuses, and number of accepted ambulances were compiled from the Residency Electronic Information System. Information on the prefectural population, urban area, and number of senior residents (postgraduate years 3–5) for specialty training was extracted from data published on the web page. The ratio of the number of first‐choice applicants to recruitment capacity (matching ratio) for each program was compared between the characteristics of the hospitals and prefectures.

**Results:**

A strong linear relationship was observed between the number of first‐choice applications and the allocated number of resident positions (correlation coefficient, .72). The matching ratio was greater in community hospitals (2.10 times compared with university hospitals; 95% confidence interval [CI], 1.75–2.53), in hospitals with higher numbers of accepted ambulance cases (1.05 times per 1000 annually; 95% CI, 1.03–1.08), and in hospitals that served a larger prefectural population (1.05 times per million; 95% CI, 1.02–1.08).

**Conclusions:**

Financial incentives do not seem to attract residency applicants. Applicants prefer non‐university hospitals located in populous areas and those that accept larger number of ambulance cases. To recruit junior residents, an emergency department may need to have higher activity with larger numbers and variety of cases.

## Graphical abstract

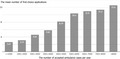

## 1 INTRODUCTION

In 2004, Japan introduced a mandatory 2‐year postgraduate training program for graduating medical students with a super‐rotation curriculum. A national matching system was established to determine the hospital residency programs best suited for the students.^1^ The goal is for residents to acquire a wide range of clinical skills with a focus on primary care. This 2‐year clinical training program is mandatory, and fixed regulations are placed on the provision of appropriate salaries and prohibition of other work during the residency. Residents can only train in university hospitals affiliated with a medical school or community teaching hospitals designated by the Ministry of Health, Labour and Welfare as clinical training facilities. Hospitals designated as clinical training facilities are required to meet various criteria; if these requirements are not met, the designation is revoked, and the facility is no longer able to accept residents. Training follows a curriculum created by the training program director and approved by the Ministry of Health, Labour and Welfare. Facilities in isolated areas (eg, clinics on remote islands), small‐ and medium‐sized hospitals and clinics, health centers, and geriatric health service facilities can be included as partner facilities of clinical training facilities.^1^

Teaching hospitals make efforts to recruit prospective residents who provide patient care, thereby representing valuable healthcare providers. Because the allocated number of resident positions is greater than the total number of applicants, there are many hospitals with smaller numbers of residents, and these hospitals are at risk of having a reduced number of healthcare providers and consequently a lower quality of care. Thus, it is important for all teaching hospitals to recruit an adequate number of residents annually.

Numerous studies have reported associations between financial incentives and specialty choices in physicians' career.^2^, ^3^, ^4^, ^5^, ^6^, ^7^, ^8^ Enari and Hashimoto analyzed data in 2006 and 2009 and reported that financial incentives affected the choice of training hospital among Japanese medical students who chose non‐university settings.^9^ In a 2006 survey, Nomura et al^10^ found that resident physicians were more satisfied with their residencies at city hospitals in terms of income, residency systems, and clinical skill training than their counterparts who performed their residencies at university hospitals. However, the findings of these studies rely on data from more than 10 years ago, calling for new analyses based on the most recent data.

The lifestyles and environment surrounding medical students have changed dramatically during the last 10 years. The most notable change in lifestyle likely is the widespread use of smartphones. Smartphones have enabled medical students to access the Internet anytime and from anywhere. Medical students now are able to collect detailed information about the clinical training hospital and amenities in surrounding areas in real time.

Since we hypothesized that young people would have a preference for large cities, such as Tokyo, Osaka, and Fukuoka, which all are easily accessible, we also surveyed whether hospitals located near a *shinkansen* (super express train) station were more popular; this point increases the novelty of this study.

Thus, we used the most updated matching data reported by the Japan Residency Matching Program (JRMP) to assess the characteristics of popular hospitals named as first choice by a greater number of graduating medical students during the matching.^11^

## 2 METHODS

### 2.1 Study design

This was a cross‐sectional study using nationwide matching data.

### 2.2 Resident matching system in Japan

The resident matching system matches candidates for postgraduate clinical training with teaching hospitals that conduct postgraduate clinical training. The combination of candidate and hospital is determined by a computer according to a certain rule (algorithm) based on the wishes of both the candidate and the hospital.^11^

### 2.3 Japan Residency Matching Program

The JRMP is composed of the Japan Medical Association,^12^ the Foundation for Promotion of Medical Training,^13^ the Association of Japan Medical Colleges,^14^ and the Association of Clinical Training.^15^ The JRMP fulfills various roles in operations related to resident matching. A website related to resident matching has been launched and provides information to participants and hospitals related to recruitment, such as the number of recruited residents for participating hospitals provided by the Ministry of Health, Labour and Welfare. A helpline has been established to answer queries about matching from participants and participating hospitals and to publicize information related to the inquiries as necessary. Schedule management, algorithm publication, and interim reports are also performed. After residency matching, surveys are conducted to improve the resident matching system.^11^

### 2.4 Data collection

Data on the numbers of applicants naming a hospital as their first choice were collected from the 2019 JRMP report on clinical training matching for junior doctors. This matching system has an interim report and a final report. Data from the interim report were used because they represented more precisely the hospitals' popularity, according to the choices of medical students in the final (6th) year.

Data on monthly salaries, bonuses, number of beds, number of accepted ambulance cases per year, and certification as a tertiary emergency center for each hospital were compiled from the Residency Electronic Information System (REIS).^16^ Information on the prefectural population, urban area (200 000 people or greater), the presence of stations for *shinkansen* (super express trains), and the number of senior residents (postgraduate years 3–5) for specialty training was extracted from data published on the web page.

### 2.5 Statistical analyses

The ratio of the number of first‐choice applicants to recruitment capacity (matching ratio) for each program was compared between the characteristics of the hospitals and prefectures. The mean numbers of first‐choice applicants were modeled with negative‐binomial model with log‐link function, including the characteristics as covariates and the log of the allocated number of residency positions as an offset variable. Correlations between multiple programs within the same hospital were adjusted through generalized estimating equations. In addition, we summarized Pearson's correlation coefficients for program‐level variables with program‐, hospital‐, and prefecture‐level variables at the program‐level data (n = 1363); correlations for hospital‐level variables with hospital‐ and prefecture‐level variables at the hospital‐level data (n = 1020); and correlations between prefecture‐level variables at the prefecture‐level data (n = 47). All analyses were conducted by SAS version 9.4 (Cary, NC, USA).

### 2.6 Ethical approval

This study was based on analysis of public data, and therefore, approval of the ethics committee was not required.

## 3 RESULTS

**Figure 1:**
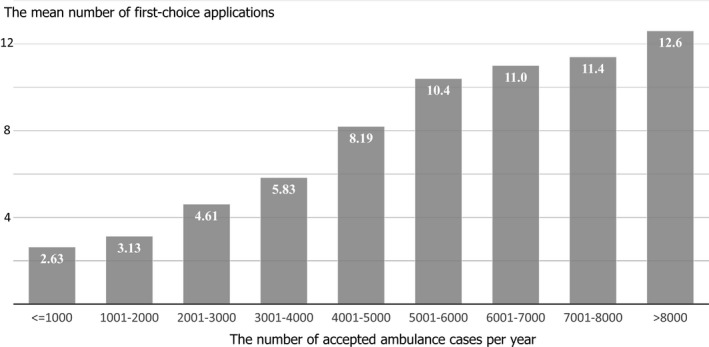
Relationship between the mean number of first‐choice applications and the number of accepted ambulance cases per year

There were 1363 residency programs in 1020 hospitals (907 community and 113 university hospitals, including affiliated hospitals). Table 1 shows the characteristics of the hospitals. A strong linear relationship (correlation coefficient, .72) was observed between the number of first‐choice applications and the allocated number of resident positions. The matching ratio was greater in community hospitals (2.10 times compared with university hospitals; 95% confidence interval [CI], 1.75–2.53) and in hospitals with higher numbers of accepted ambulance cases (1.05 times per 1000 annually; 95% CI, 1.03–1.08). Figure 1 shows the relationship between the mean number of first‐choice applications and the number of accepted ambulance cases. In addition, the matching ratio was greater in hospitals that served a larger prefectural population (1.05 times per million; 95% CI, 1.02–1.08) (Table 2). There were no significant associations between the number of first‐choice applications and the location of the hospital near stations for *shinkansen* (super express trains), residents' salaries, or number of hospital beds.

**Table 1:** Demographic and program‐matching data

Variable	Median (1st–3rd quartile)	n (%)
Program level (n = 1363)		
Number of first‐choice applicants	4 (1–9)	—
Recruitment capacity	5 (2–10)	—
Monthly salary (yen)	320 300 (300 000–380 000)	—
Bonus given (n = 1339)	—	580 (43.3)
Hospital level (n = 1020)		
University hospital	—	113 (11.1)
City hospital	—	907 (88.9)
Urban area (≥200 000 people)	—	575 (56.4)
*Shinkansen* (super express train) station	—	311 (30.5)
Number of hospital beds (n = 1005)	402 (311–535)	—
Annual number of accepted ambulance cases (n = 1005)	3293 (2033–4965)	—
Tertiary emergency care (n = 1005)	—	290 (28.9)
Prefecture level (n = 47)		
Number of hired senior residents	94 (63–146)	—
Population	1 648 177 (1 113 980–2 727 172)	—

**Table 2:** Factors related to first choice of clinical training facilities (multilevel model)

Variable	Ratio of matching ratios^a^	95% confidence interval		*P*‐value
Program level				
Salary per 100 000 yen	0.99	0.93	1.06	.83
Bonus	1.06	0.97	1.15	.19
Hospital level				
City vs university hospital (including branch hospitals)	2.1	1.75	2.53	<.001
Urban area (≥200 000 people)	1.09	0.99	1.19	.07
*Shinkansen* (super express train) station	1.08	0.99	1.18	.10
Number of hospital beds (per 100)	1.00	0.97	1.03	.90
Annual number of accepted ambulance cases (per 1000)	1.05	1.03	1.08	<.001
Tertiary emergency care	1.04	0.94	1.16	.40
Prefecture level				
Number of hired senior residents (per 10)	1.000	0.998	1.002	.78
Population (per 1 million)	1.05	1.02	1.08	<.001

^a^ The matching ratio is defined as the ratio of the number of first‐choice applications to the recruitment capacity number. In the negative‐binomial log‐link regression models for first‐choice application number, including log (recruitment capacity number) as an offset variable whose coefficient was set at 1, each exponentiated coefficient is interpretable as the ratio of matching ratios between distinct levels of that variable. The model was fitted by generalized estimating equations using hospitals as clusters

Despite the strong correlation (.9) between two prefecture‐level variables (Table 3), we confirmed that including or excluding these variables from the regressors did not affect the coefficients of other variables. We also assessed the dependence between the variables using a variance inflation factor (without considering the nonlinear model form for matching ratio and the multilevel structure) at the program‐level model fit. The values ranged from 1.3 to 2.5 for the program‐ and hospital‐level variables and approximately 6.5 for the prefecture‐level variables.

**Table 3:** Correlation between variables

Variable		Variable									
		A	B	C	D	E	F	G	H	I	J
Program‐level correlation (n = 1363)											
A	Salary	1.000	0.066	0.453	−0.293	−0.118	−0.433	−0.275	−0.310	−0.307	−0.296
B	Bonus		1.000	0.402	−0.086	0.059	−0.327	0.017	−0.236	−0.130	−0.100
Hospital‐level correlation (n = 1020)											
C	City hospital vs university hospital			1.000	−0.102	−0.010	−0.518	−0.025	−0.316	−0.107	−0.091
D	Urban area				1.000	0.388	0.196	0.199	0.052	0.228	0.251
E	Shinkansen (bullet trains) station					1.000	0.076	0.068	0.021	−0.064	−0.039
F	Number of hospital bed						1.000	0.451	0.567	0.121	0.135
G	Annual number of times ambulances							1.000	0.416	0.247	0.336
H	Tertiary emergency care								1.000	−0.012	−0.012
Prefecture‐level correlation (n = 47)											
I	Number of hired senior resident									1.000	0.899
J	Population by prefecture										1.000

## 4 DISCUSSION

This study is the report from Japan to examine the characteristics of teaching hospitals that are popular choices by medical students using the most updated matching data reported by the JRMP. Community hospitals (vs university hospitals), increased acceptance of ambulance cases, and larger prefectural populations were significant attributes that appealed to medical students in the final (6th) year who were selecting a teaching hospital.

University hospitals are considered medical research institutes in Japan. They are unable to provide adequate primary and general healthcare training opportunities, leading to lower popularity among junior (postgraduate years 1 and 2) residents. Moreover, emergency care training is pivotal to a hospital's popularity among students selecting a residency program, regardless of its status as a tertiary emergency center. Japanese hospital physicians of all specialties are required to examine patients in emergency rooms. Thus, medical students consider hospitals with a greater number of emergency patients as superior training centers.

In 2016, Mizuno et al^17^ reported the performance of 2015 junior resident physicians (postgraduate years 1 and 2) working in 208 Japanese hospitals nationwide in the General Medicine In‐Training Examination (GM‐ITE). GM‐ITE scores were generally higher for residents working at training sites with a greater volume of patient admissions and a lower number of nonresident physicians. The findings of this study suggested that experience with many patients, despite a limited availability of mentors, could have higher educational value for junior clinical trainees.

Our analysis revealed that training facilities with higher allocated numbers of resident positions were more popular among applicants. This may reflect conservative Japanese values, including impressions of higher emotional support and security in an environment with more colleagues who enter at the same time, and a lighter expected per‐resident workload from the availability of more residents for allocation of diverse incidental tasks associated with training.

Hospitals that are popular among students selecting a residency tend to be located in areas of high population. This finding is congruent with the general trend demonstrated by young Japanese individuals preferring to live in urban locales. In addition to the comforts of urban amenities, the reasons for the popularity of areas with higher populations may include having more opportunities for out‐of‐hospital learning. A previous study reported that participation in workshops contributed to increasing basic clinical competency.^18^, ^19^, ^20^, ^21^ Proximity to a *shinkansen* station may not be a significant factor for popularity of a residency program, although it is generally preferred by young people as regards their choice of workplaces. Residents in initial training are generally very busy^22^ and do not place value on accessibility for personal domestic long‐distance travel.

Salaries and bonuses were also unimportant factors for choice of residency. This is consistent with the belief that financial incentives do not improve the quality of medical care.^23^, ^24^, ^25^, ^26^ A 2‐year training period may seem short, and therefore, salary is not a crucial factor for selecting a training hospital. In their previous Japanese study, Enari and Hashimoto reported that financial incentives influenced medical students' choice of hospitals for residency;^9^ however, this finding was not supported by our data. This can be explained by the following two reasons. The first is the change in the times. As stated in the Introduction, the lifestyles and environments surrounding medical students have changed dramatically during the last 10 years. The most notable change has involved the markedly increased facility of collecting information. Second, the results may be attributed to analysis models and analyzed data. They mainly reported the estimates of a linear fixed‐effect model fitted to repeatedly measured data (2006 and 2009). The fixed‐effect model could adjust for all unmeasured variables within the programs that were not changed from 2006 to 2009, but not for the variables that changed along with the salary during the period. On the other hand, our analysis used cross‐sectional data and explicitly adjusted for the measured program‐, hospital‐, and prefecture‐level variables by using the log‐linear model conditional on the number of recruitment capacity (as an offset). We cannot judge theoretically or empirically which of the apparently conflicting results are more reliable than the other. Rather, they may reflect the distinction between the research questions; we explored the cross‐sectional association between financial incentives and students' choice of the programs, while Enari and Hashimoto might have pursued the impact of financial incentives on the choice within the same programs.

This study has several limitations. First, we were not able to collect data on the students' home towns. Students may select hospitals that are close to their parents' homes or their own homes for their residency. Second, we did not include whether the hospital had a general medicine department in our analysis. Because junior clinical training aims for the acquisition of a wide range of clinical skills, with a focus on primary care, it is of great importance whether the facility has a department of general medicine for applicants selecting their residency sites. Third, we did not assess whether the facilities had mentors who were foreign nationals; this could be an important factor for residents who wish to do their residency under the mentorship of foreign‐national physicians to develop clinical skills with global standards. Fourth, we focused on data from 2019 because we specifically wanted the results of our analysis to reflect the most recent data. This not only allowed us to analyze the newest data, but also limited our study to analysis of the data from a single year. Finally, we primarily selected the factors that we deemed important among the data on clinical training hospitals accessible within the REIS. However, factors other than these may influence popularity among medical students, such as the number of supervising physicians at each training hospital, online medical resource accessibility, and frequency of lectures related to the primary care. It is another limitation of our study that we did not assess.

In conclusion, financial incentives do not seem to attract residency applicants. Applicants prefer non‐university hospitals located in populous areas and those that accept larger numbers of ambulance cases. To recruit junior residents, an emergency department may need to have higher activity with larger numbers and variety of cases.

## CONFLICT OF INTEREST

None.
